# Neuron-Glia-Immune Triad and Cortico-Limbic System in Pathology of Pain

**DOI:** 10.3390/cells10061553

**Published:** 2021-06-19

**Authors:** Isabella Murray, Gayatri Bhanot, Aditi Bhargava

**Affiliations:** 1 Department of Obstetrics and Gynecology, Center for Reproductive Sciences, University of California San Francisco, San Francisco, CA 94143, USA; isabella.murray04@icloud.com (I.M.); gbhanot@ucsd.edu (G.B.); 2 Eleanor Roosevelt College, University of California San Diego, San Diego, CA 92122, USA

**Keywords:** central nervous system, dorsal root ganglia, gliopathic pain, mental health, pain perception

## Abstract

Pain is an unpleasant sensation that alerts one to the presence of obnoxious stimuli or sensations. These stimuli are transferred by sensory neurons to the dorsal root ganglia-spinal cord and finally to the brain. Glial cells in the peripheral nervous system, astrocytes in the brain, dorsal root ganglia, and immune cells all contribute to the development, maintenance, and resolution of pain. Both innate and adaptive immune responses modulate pain perception and behavior. Neutrophils, microglial, and T cell activation, essential components of the innate and adaptive immune responses, can play both excitatory and inhibitory roles and are involved in the transition from acute to chronic pain. Immune responses may also exacerbate pain perception by modulating the function of the cortical-limbic brain regions involved in behavioral and emotional responses. The link between an emotional state and pain perception is larger than what is widely acknowledged. In positive psychological states, perception of pain along with other somatic symptoms decreases, whereas in negative psychological states, these symptoms may worsen. Sex differences in mechanisms of pain perception are not well studied. In this review, we highlight what is known, controversies, and the gaps in this field.

## 1. Introduction

**Figure 1:**
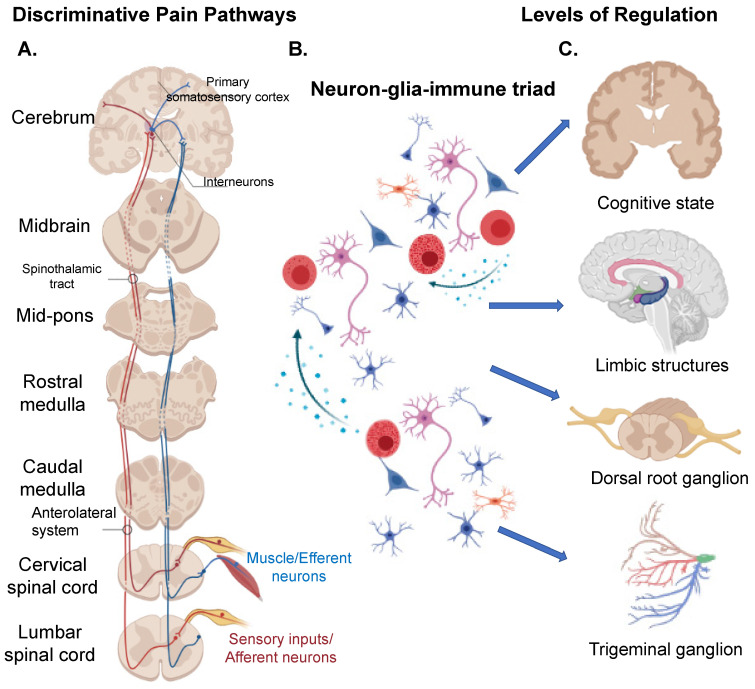
Pain pathways and levels of regulation. (**A**) The pain signal cascades through the central and peripheral nervous systems. Noxious sensory inputs from the lower and upper parts of the body are sensed by the afferent neurons that feed to the lumbar and the cervical spinal cord divisions, respectively. The information is relayed to the primary somatosensory cortex of the cerebrum; interneurons in the CNS transmit information to the efferent neurons that relay signals from the CNS to the effector organs, such as the muscles and glands. (**B**) The neurons in the CNS or the PNS are surrounded by glial cells. Resident or infiltrated immune cells release mediators that in turn modulate glial and neurons activity function at various levels. (**C**) Pain signal is regulated at several levels; an individual’s cognitive state regulates pain perception. The limbic system is key for integrating pain perception. Immune and glial cells respond to injury or noxious stimuli and modulate function of dorsal root ganglia, trigeminal ganglion, and limbic structures to alter pain perception. All these inputs are integrated and together constitute one’s cognitive state, which can affect pain perception and experience. Pain can be regulated at each level via the mediators summarized in Table 1.

Pain is a complex concept that requires the orchestration of a plethora of structures and mechanistic pathways. Sensory-discriminative and cognitive-affective systems are yin-yang of brain networks that process pain. The degree of pain perceived largely remains a subjective or qualitative process, with “objective” or quantitative markers of pain remaining elusive. Both central and peripheral structures play a part in this complex, highly coordinated process; from the sensory neurons and dorsal root ganglia that serve as conduits, to neurotransmitters responsible for transmitting information from neuron to neuron to signal pain, and glia/support cells that help in detoxification and extinguishing processes (Figure 1 and Table 1). Nociceptive pain resulting from physical damage may be caused by thermal (heat or cold) or pressure (pinch, prick, etc.) factors and is sensed by nociceptive receptors on sensory afferent neurons that evoke action potentials that are transferred via the spinal cord to the brain. Clinical pain, for example, due to the result of nerve injury (neuropathic pain) is often enhanced in the presence of otherwise non-noxious stimuli (allodynia) or stimuli that are known to evoke pain (hyperalgesia). Development of neuropathic and musculoskeletal pain involves damage to the neurons, various cell types in the dorsal root ganglion, as well as recruitment of peripheral immune cells, referred to as the neuron-glial-immune triad.

Cognitive processes and expectations are contributors to pain perception. For example, wisdom teeth extraction is expected to be a painful and traumatic experience; the more a person dwells on the impending procedure, the more traumatic and painful the experience. Trust in capabilities, dexterity, and reviews about the physician performing the procedure can change one’s expectation, and hence pain experience and perception. Similarly, the placebo effect is powerful and well-known [1]. Countless studies have shown that placebo pills, expectations, and conditioning can resolve mild-to-severe pain experience in various settings [2,3,4,5,6,7], a concept that defies logic and seems hard to fathom when looking at how complex the mechanisms behind pain and perception are. However, mechanisms that underlie placebo effects remain unelucidated and controversial.

Approximately 1.7 billion people globally and one in two adults in the United States suffer from musculoskeletal pain [8]. While lower back pain is the most common type of musculoskeletal pain afflicting people worldwide, osteoarthritis, tendonitis, (fibro)myalgia, and stress fractures are other common pain categories. Musculoskeletal pain can be localized or widespread, affecting nerves, bones, muscles, tendons, and ligaments. It can be acute or chronic and manifests with a variety of symptoms. Bone pain can be deep, penetrating, or dull, whereas muscle pain is often less intense, but both can be debilitating and result in a decreased quality of life. Tendon, ligament, and joint pains range from mild to severe. Regardless of the pain category or cause, there are individual differences in perception, coping, and behavioral adaptations. Pain management clinical guidelines recommend a multimodal approach balancing patient safety, comfort, physical, cognitive, and pharmaceutical approaches [9].

### 1.1. “Central” Structures Orchestrating Pain

The lateral thalamus and somatosensory cortex of the central nervous system (CNS) process nociceptive input received from the periphery, including its intensity, localization, and quality, and constitute the sensory-discriminative system. The anterior insula and the anterior cingulate cortex, are involved in sensing the psychological aspects of pain, and together with the corticolimbic system comprise the cognitive-affective system (Figure 1). Other CNS regions associated with both pain and emotional aspect of pain perception include the medial prefrontal cortex, the nucleus accumbens, and the amygdala, and other parts of the corticolimbic system. Since the functional circuitry between these regions varies between individuals, these differences influence and may explain nuanced cognitive-emotional states, pain perception, and experience between individuals with similar injuries or trauma.

### 1.2. Peripheral Nervous System (PNS)

Providing our body with the ability to move our limbs and feel sunlight, the PNS plays an important role in sensing the many inputs we encounter every day. The afferent (or sensory) component of the PNS is responsible for relaying information such as sensation and perception to the CNS. The efferent (or motor) component brings information from the CNS to the PNS, specifically the muscles (Figure 1). The autonomic, sympathetic, parasympathetic, and enteric nervous systems are all components of the PNS along with the trigeminal, cranial, peripheral, and spinal nerves with their roots and rami. Sensory fibers (originating from endings in skin, muscles, tendons, periosteum, bones, and joint), motor fibers (terminating in end plates in skeletal muscle), efferent autonomic fibers (innervating blood vessels, sweat glands, and arrectores pilarum muscle), and visceral afferent fibers, together make up the peripheral nerves. Afferent fibers in skin and dura mater located near blood vessels have a leaky blood-brain barrier (BBB), thus they are more vulnerable to neurotoxic substances, providing an explanation as to why chemotherapy may cause neuropathic pain. Understanding the mechanisms by which the nervous system processes external stimulation provides insight into how our bodies sense and process pain (Figure 1).

### 1.3. Neuron Clusters/Ganglion Systems in Pain

The dorsal root ganglion (DRG) consists of pseudo-unipolar neurons, in which a single axon bifurcates into two separate branches, allowing for distal and proximal processes [10]. They transmit sensory information from thermoreceptors, proprioceptors, nociceptors, chemoreceptors, and more to the CNS. Interactions between soma of DRGs are limited by layers of satellite glial cells (SGCs) between them. These SGCs express receptors for molecules involved in neurological signaling, such as cytokines, glutamate transporters, ATP, bradykinins, and more [11]. DRGs change morphologically following injury, altering the DRG function and pain perception/threshold [12,13,14]. The DRG plays an active role in both acute and chronic pain. Since the majority of first-order neurons lay in the DRG, sensation, notably nociception, is regulated by the DRG before passing to the brain. In DRG neurons, G protein-coupled receptors, which are associated with nociception, are expressed. Compressing the DRG manually lowers the action potential threshold, therefore making action potentials more likely to fire, even after removal of the stimulus [15]. C fibers in the DRG interact with thermomechanical reception and contain several peptidergic modulators such as Substance P and calcitonin gene-related peptide [16,17,18]. DRG neuron damage is associated with chronic pain conditions and neuropathies, in part due to the releasing of these mediators [19]. Emerging evidence that involves metabolic changes in DRGs suggests a more active rather than passive role in the pain process [15]. The DRG has a permeable blood-brain barrier, rendering it more vulnerable to neurotoxic substances and metabolites in the blood. In addition to being a center for pain processing, the DRG size and location along with SCGs make for an attractive pain treatment target [11,15].

Trigeminal ganglion is the largest sensory ganglion which transmits information from the head, face, and the mandibles (jaws) to the brain. The trigeminal has three branches: ophthalmic, maxillary, and mandibular. Afferent nerve fibers originating from the trigeminal ganglion innervate the cerebra dura mater [20], whereas those from the tongue feed into the trigeminal and hypoglossal nerves. Like DRGs, the trigeminal is also shielded by SGCs. Damage to any of the trigeminal branches is the root cause of orofacial pain, such as headaches, myofascial, or tooth pain [21]. The suppression of glial gap junction protein, connexin43 or glutamic acid decarboxlylase, the enzyme responsible for synthesizing GABA in SGCs of rats with infraoribital/maxillary nerve injury, reduces pain-like behavior and suggests that GABA acts at the trigeminal ganglion, not at the spinal level [11,22]. Paradoxically, suppression of connexin43, potassium channels Kir3 and Kir4.1 in SGCs of uninjured rats increases pain-like behavior. In rats, orofacial pain after injury to the trigeminal nerve is alleviated after the suppression of sodium channel Na_v_1.7 or, or other purinergic receptors. In humans, carbamazepine is often used to treat trigeminal neuralgia, but it has several severe side-effects.

### 1.4. Glial/Non-Neuronal Cells in Pain

Several non-neuronal cell types are key in ensuring neuronal function. In fact, isolated neurons do not behave or function the same as they do when surrounded by support cells that include the glia, astrocytes, and Schwann cells.

#### 1.4.1. Glial Cells

Glial cells in both the CNS and PNS play vital roles in the development and maintenance of pathological pain. The classification of CNS/PNS glial cells is based on their location and morphology, and they often express common markers. Injured or dysfunctional CNS glial cells contribute to several neurodegenerative disorders [23]. Although neuronal cells have more traditionally been targeted in therapies for chronic pain, emerging evidence has been pointing to the virtue of targeting glial cells as well. Models of chronic pain have revealed that SGCs, astrocytes, oligodendrocytes, and Schwann cells all proliferate, undergo phenotypical changes, and release mediators upon injury [11,12,24].

#### 1.4.2. Satellite Glial Cells

Satellite glial cells surround neurons in the sensory ganglia of the PNS. Upon nerve injury, SGCs themselves are not often directly damaged [12]. Rather, they respond to the damage done to the injured axons of neurons. The indirectness of this response implies active communication between neurons and SGCs that can lead to pathological pain. One form of molecular signaling implicated in the pain process is glutamate, the primary excitatory neurotransmitter of the nervous system. One role of the SGCs is to uptake extracellular glutamate released by neurons [11]. When this uptake process is hindered by changes brought on by injury, more extracellular glutamate may remain and continue to stimulate nociceptive neurons. Excess extracellular glutamate contributes to chronic pain [11,25]. Additionally, ATP released by neurons upon injury has stimulatory effects in multiple locations [12]. In SGCs, ATP release activates specific purinergic receptors. ATP can also trigger the secretion of pro-inflammatory cytokines from SGCs, which are already known to be implicated in pain [26].

#### 1.4.3. Astrocytes

Astrocytes, the most abundant glial cells in the CNS, are regulators of chronic pain. Astrocytes are activated by injury and are classified as A1 or A2 astrocytes [27]. A1 astrocytes are toxic and promote the death of injured neural cells. A2 astrocytes are neuroprotective and promote healing. This differentiation of astrocytes, called “astrogliosis,” has both beneficial effects when it comes to healing and the immune response, but also contributes to the development and maintenance of chronic pain. Another subpopulation of astrocytes, B1 and B2 adult neural stem cells, with unknown function have been described. These B cells are ciliated, contact blood vessels, and have high levels of ribosome synthesis [28]. Astrocyte activation observed in models of pain is associated with pain behavior following nerve damage [29]. Compared to microglia, astrocytic activation tends to occur later after the injury; activated astroctyes migrate to the injury site, proliferate, and induce scar formation via activation of the integrin-N-cadherin pathway [30]. Inhibition of astrocyte activation can reverse or reduce chronic pain [29]. However, this leaves the astrocyte-deficient organism more vulnerable, due to a reduced immune and tissue repair capacity. Further research is needed into ways to target only the subsets of astrocytes implicated in the pain response, while still allowing for a functional immune function.

#### 1.4.4. Schwann Cells

Similar to SGCs and astrocytes, Schwann cells release important mediators that interact with neurons and that are implicated in chronic pain. Schwann cells, myelinate neurons in the PNS, are some of the initial cellular detectors of injury [31]. Blocking specific receptor function in Schwann cells reduces pain [32]. Nerve regeneration is faster in mice with non-functional Schwann cells after sciatic nerve crush injury, but these mice display other abnormalities as well as develop neuropathic pain. This suggests that abnormalities in Schwann cells can contribute to the development of chronic pain following injury [33]. Long non-coding RNA, H19, which is maternally expressed, is upregulated in Schwann cells following nerve injury [34]; H19 codes for miR-675 and inhibits let-7a/b to modulate expression of several proinflammatory cytokines, that in turn modulate pain.

### 1.5. Immune Cells in Pain

The immune system has long been recognized for its role in the generation and maintenance of pain, with mechanisms ranging from cytokine release to inflammation [35]. The role of resident versus infiltrated immune cells has not been as widely explored. Understanding the changes some of these innate immune cells undergo upon injury, as well as associated impacts on pain and sensitivity, may lead to further treatments for chronic pain.

#### 1.5.1. Microglia

Microglia are the resident innate phagocytic immune cells of the CNS. Microglia undergo microgliosis, resulting in morphological changes, express specific markers such as CD45, CD11b, transmembrane protein 119, and purinergic receptors [36]. In normal physiological state, microglia are quiescent but are actively involved in surveillance. In the presence of abnormal signals from neurons or other glial cells, microglia undergo morphological and functional changes to become activated. Exogenous activating signals include, but are not limited to non-self genetic material from bacteria and viruses, bacterial lipopolysaccharides, and pathogen-associated molecular patterns (PAMPs). Examples of endogenous signals are protein aggregates such as plaques or amyloids that can be released by other glial cells or danger/damage-associated molecular patterns (DAMPs) [36]. Activation of M1 class microglia is in response to injury and sustained activation leads to cytotoxicity, as it is associated with the robust activation of proinflammatory cytokines. Activation of M2 class migroglia dampens inflammation and promotes recovery and homeostasis. Thus, microlgia are implicated in the initiation of mechanical allodynia rather than chronic pain. Cytokines are potent mediators of neuron-glial function [37]. Microglial activation in chronic constriction injury of the sciatic nerve in mice is associated with chronic mechanical hyperalgesia, along with depression-like behavior. Chronic constriction injury also caused increases in soma size and microglial number in the medial prefrontal cortex, hippocampus, and amygdala eight weeks post-injury in mice. Additionally, genes associated with microglial activation or depression-like behavior were upregulated in these brain areas, suggesting post-injury microglial activation at delayed time points, and indicates microglial activation in certain chronic pain-related disorders [38].

#### 1.5.2. Natural Killer Cells

NK cells are cytotoxic immune cells mediating innate immune response, although roles for NK cells in pain are equivocal. In mice, loss of sensation, due to degeneration of injured afferents, is associated with NK cell function [39]. Patients with musculoskeletal pain have a lower percentage of NK cells in their peripheral blood than healthy patients, although pain relief does not restore NK cell numbers [40].

#### 1.5.3. Neutrophils

Neutrophils are reported to activate and sensitize sensory neurons, which in turn release mediators that activate neutrophils [41]. Release of IL-1β causes neutrophils to release reactive oxygen species or prostaglandins leading to hypernociception [41,42].

#### 1.5.4. Helper T Cells

T cell subsets produce either pro and anti-inflammatory cytokines and thus can have both an excitatory and inhibitory role in chronic pain. Emerging data from clinical studies suggests that in patients with certain chronic pain conditions, there are phenotypical changes in certain subsets of their T cells [43]. In rats, chronic pain caused epigenetic changes in T cells [44]. T cells can influence transition from acute to chronic pain, although therapeutics that aim to specifically block the effects of pain-promoting T cell subsets are not easy to develop.

#### 1.5.5. Mast Cells

Mast cells are found throughout the body and are primarily involved in the inflammatory response. Upon activation resulting from injury, mast cells release mediators to evoke innate immune responses. Some of these mediators, such as histamine (which stimulates A-delta and C fibers), tryptase (which leads to nociceptor activation), and nerve growth factor (which stimulates pro-nociceptive mediators) have described roles in pain. When these mediators communicate with nociceptors on sensory nerve fibers, the fibers release inflammatory neuropeptides. This creates a positive feedback loop of inflammation and pain, because the peptides only further stimulate the mast cells to release more mediators [45]. Mediators released by mast cells also have more indirect effects. Pain mediators such as prostaglandins and cytokines are impacted by mast cell releases, indicating that mast cell dysregulation can lead to chronic overstimulation and hyperalgesia. The release of neurotransmitters such as Substance P and glutamate by neurons sensitized by mast cells also contributes to pain. Other components of the pain response regulated by mast cells include microglia, which can be activated by mast cell mediators. Meningeal mast cells secrete mediators that act in endocrine (both paracrine or autocrine) manner and contribute to inflammation associated with migraine pain [46]. Mucosal mast cells also modulate visceral hypersensitivity and gut function in humans and in animal models of functional bowel diseases [47,48,49,50].

### 1.6. The Neuron-Glial-Immune Triad Interaction in Pain Pathology

Disorders that involve demylination of the neurons such as the Guillain-Barre’syndrome and Amyotrophic lateral sclerosis (ALS) involve overactive T cells that are unable to distinguish self from non-self in the initial phase. Both T and B cells are involved in late stages of these syndromes. Nerve and muscoloskeletal pain associated with Guillain-Barre´syndrome and ALS is often hard to control and no single pain treatment modality is effective. In peripheral neuropathic pain, neutrophil that infiltrate to the nerve injury sites in the acute phase release nerve growth factor and chemokines. Chemokines harboring the C-C and C-X-C motifs in turn recruit resident macrophages as well as monocytes from the peripheral blood acting via cytokine receptors CCR1, 2, and 5 [51]. Activated immune cells in concert with injured glial cells and denervated Schwann cells secrete matrix metalloproteases that damage the basal lamina [52] of endothelial cells of blood vessels lining the BBB, making it leaky and permeable to substances that otherwise do not cross a healthy BBB. Vasoactive peptide hormones such as calcitonin gene-related peptide (CGRP), Substance P, corticotropin-releasing factor, urocortins, bradykinin, and other mediators such as nitric oxide are either released from the damaged neurons, endothelial cells, or immune cells surrounding the damaged area to cause edema, swelling, and hyperemia. Injury-induced inflammation can cause CD31+ macrophages and leukocytes to infiltrate the vasculature making it permeable and leaky [53].

Animal models of pain reveal that the expression of genes regulating immune cell function are upregulated in DRG after neuron injury [54]. These gene products facilitate the recruitment of macrophages and T cells, which in turn secrete cytokines such as IL-6, TNF-α, IL-1β that modulate action potentials of neurons. Blocking IL-6 or IL-1 signaling in animal models of pain inhibits neuropathic-pain-like behaviors [55,56]. Microglia are recruited in both the dorsal and ventral horns of the spinal cord in the acute phase of injury; both resident and circulating immune cells are involved. Microglia express receptors for C-C motif cytokines suggesting communication between immune cells and injured afferent fibers. Activation of microglia causes increased release of protease Cathepsin from the lysosomes to enhance neuropathic pain [57]; these proteases might also be involved in pain associated with pancreatitis, a disease in which Cathepsins and protease play an important role. Inhibition of Cathepsin in spinal microglia ameliorates pain [57]. Discussion of cancer- and chemotherapy-related chronic pain is beyond the scope of this review, but glial cells play an important role, as demonstrated using animal models [58,59,60].

### 1.7. The Cortico-Limbic System in Pain

The top-down processing nature of pain in the brain supplements the way an emotional state influences pain perception. The dorsolateral prefrontal cortex, rostral anterior cingulate cortex, and the periaqueductal gray are all associated with pain relief; these structures also partake in different cognitive alterations, or mental states. Further, the top-down processing connects back to possibly alter the responses elicited in the dorsal root ganglion. Emotional states can not only alter the way the pain is perceived but changes the structure of the brain itself in the process [61]. Chronic pain symptoms are also exacerbated by negative cognitive and emotional responses to the initial pain or physical trauma [62]. Chronic pain is connected with the hippocampus, a limbic structure associated with memory; hippocampal neurogenesis may become modulated in ways that make the memories of pain unforgettable. Fear conditioning is highly associated with the amygdala region of the limbic system, bringing pain and memory into the equation. The pathway in which pain is processed in the amygdala differs from other nociceptive inputs, demonstrating a hemispheric lateralization in processing [63]. Working together, the hippocampus and amygdala form long-term memories, specifically those including heavy emotions. Initial pain is associated with an abundance of emotions, explaining why it often is mistaken for a long-term emotional memory the brain must store. This then translates to the upkeep of chronic pain, explaining the role the limbic system has in perpetuating the symptoms [62].

### 1.8. Mental Health/Altered Cognitive States in Pain Perception

The link between an emotional state and the way pain is experienced proves to be larger than what is widely acknowledged. Positive, negative, and other altered emotional states translate to changes in perception of other stimuli, including pain. In positive psychological states, perception of pain decreases, along with other somatic symptoms, and in negative psychological states, these symptoms often worsen. Furthermore, pain and an individual’s psychological state are inter-dependent, where high pain intensity can decrease emotional well-being and further exacerbate pain perception. This cyclical relationship explains why maintaining good mental health carries through all parts of the body, especially when feeling pain. The role of mental and emotional well-being has slowly come to light as an essential part in the way humans perceive and experience pain [64].

The relationship between pain and a cognitive state has been demonstrated in a multitude of patients. Patients with psychiatric disorders such as schizophrenia, depression, and/or anxiety, often develop chronic pain, in turn worsening symptoms of their preexisting psychiatric disorders; or, patients with chronic pain are at increased risk of developing mood disorders. This cyclical relationship can be attributed to numerous factors, from the decrease quality of life that comes with chronic pain, to the stress the body is under from chronic pain exacerbating disorderly symptoms. Further, physical exercise, that alleviates pain and emotional distress is often harder to perform by patients with chronic pain, due to physical limitations. A greater acquisition of conditioned fear is reported in women compared to men with posttraumatic stress disorder [65]. The growing body of evidence suggests that leaky BBB may allow peripheral cytokines to cross that barrier in mental disorders. Metabolites of tryptophan such as kynurenine also cause inflammation in the brain [64] and may alter action potentials. Developing effective cognitive therapies for treating patients with chronic pain is an attractive alternative.

Imaging studies have demonstrated association between chronic pain and structural changes in certain brain regions; the longer an individual suffers from chronic pain, the more pronounced these changes are. The results of many brain imaging studies on patients with chronic pain suggest the structural changes of many parts of the limbic system, including the hippocampus, amygdala, nucleus accumbens, and periaqueductal grey matter. Structural changes such as these could explain the link between emotional distress and pain [66]. When the body senses pain, the corticolimbic system remains active, while the pain processing transitions from the sensory systems to emotional regions [62]. Catastrophizing has been noted to be the most linked cognitive response that invoked change within the subject’s brains. Although the directionality of the associations cannot be concluded, increased brain activity in patients who experience pain catastrophizing is clearly present. Furthermore, the links to network changes can be observed in all cognitive alterations, demonstrating that even when the brain is in resting state, these cognitive alterations have an effect on function [67]. With further research on how these different cognitive alterations impact specific groups of people, including different genders, sex, age, and other socioeconomic and environmental influences, steps can be taken to combat both chronic pain and poor mental health.

## 2. Sex Differences in Injuries and Pain Perception—An Emerging Area

Sex differences are reported in the incidence and prevalence of musculoskeletal pain. Studies from healthy adult volunteers who underwent experimental pain to identify pressure pain threshold did not find any sex differences in muscle fatigue times between men and women, although the attitude towards pain differed between the sexes [68,69]. Anatomical differences may explain sex differences in risk towards certain injuries and associated pain [70], although the mechanisms behind these sex differences are not known. Sex-specific differences in immune and stress responses [71,72] can be one explanation for differences in chronic pain conditions in men and women. Sex differences are reported in subsets of circulating T cell populations in men and women; higher CD4+ helper T cell counts and higher CD4:CD8 ratios are reported in women compared with men. Men have higher NK cell frequencies than women. Phagocytic activity tends to be higher in neutrophils and macrophages from men than those from women. Sex differences in resident or lymphatic immune cells in pain has not been reported. These differences may help explain discrepancies in chronic pain conditions [73]. In men with chronic pelvic pain, increased amounts of the mast cell mediators tryptase and nerve growth factor is reported [74]. Altogether, this evidence points to the contributions of mast cells to the development and maintenance of pain, and to their involvement in the transition of acute to chronic pain. Rodent studies have revealed significant sexually dimorphic differences in microglia number and phenotype. Sex differences in the numbers of microglia in the preoptic area, hippocampus, parietal cortex, and amygdala are reported. In the preoptic area, the microglia of male rats have appeared more “activated” due to their larger size and decreased length and branching [75]. Astrocytic morphology tends to be more stellate in male mice, and bipolar in female mice. The presence of different sex steroid hormones affects how astrocytes respond to glutamate, which is vital for signaling [76]. After injury, astrogliosis is more prominent in male rodents than in female mice [77].

## 3. Conclusions

In conclusion, pain perception is a complex process with several levels of regulation (Figure 1) and numerous neurotransmitters and modulators (Table 1). Cytokines, interleukins, purinergic, peptidergic, cannabinoid receptors, sodium, and potassium channels, MAP kinases, proteases have all been targets of pain therapy. Glial and support cells are attractive targets, as they are involved in both the initiation as well as extinguishing signals that modulate pain. There is no panacea for pain nor one particular treatment can resolve all types of pain. Hence, a combination therapy is probably best, as targeted therapies have not yet proven effective. While sex differences in pain incidence are reported, mechanisms behind these sex differences remain understudied.

## Figures and Tables

**Table 1:** Neurotransmitters and neuromodulators of the central, peripheral, and enteric nervous systems.

Neurotransmitter and Neuromodulators
Adenosine Triphosphate (ATP)
Algogen, Bradykinin (BK) inflammatory mediator
Amino Acids (Includes glutamate: most abundant excitatory neurotransmitter, Serine, Glycine, etc.)
Artemin and glial cell-line derived neurotrophic factors (GDNF)
Brain-derived nerve growth factor (BDNF)
Calcitonin Gene-Related Peptide (CGRP)
Cytokines and chemokines
Gamma aminobutyric acid (GABA)
Nerve Growth Factor
Neuropeptides (includes corticotropin-releasing hormone, urocortins 1–3)
Norepinephrine
Tachykinins (includes Substance P, neurokinin A, neurokinin B)

## Data Availability

No new data were generated in this study. Data sharing is not applicable to this article.
